# Laboratory Diagnosis of Hendra and Nipah: Two Emerging Zoonotic Diseases with One Health Significance

**DOI:** 10.3390/v17071003

**Published:** 2025-07-17

**Authors:** Shaun van den Hurk, Aurelle Yondo, Binu T. Velayudhan

**Affiliations:** Athens Veterinary Diagnostic Laboratory, College of Veterinary Medicine, University of Georgia, Athens, GA 30602, USA

**Keywords:** Hendra virus, Nipah virus, zoonosis, diagnostic techniques

## Abstract

Hendra virus (HeV) and Nipah virus (NiV) are two highly pathogenic RNA viruses with zoonotic potential, which can cause severe diseases with high mortality rates (50–100%) in humans and animals. Given this context, these viruses are classified as Biosafety Level 4 (BSL-4) pathogens, thus limiting research studies. Despite the high case fatalities, there are currently no human vaccines available for either virus, owing in part to the limitations in research and hesitancy in funding. In the absence of widespread vaccination, diagnostic tests are crucial for the rapid identification of cases and disease surveillance. This review synthesizes current knowledge on the epidemiology, transmission dynamics, and pathogenesis of NiV and HeV to contextualize a detailed assessment of the available diagnostic tools. We examined molecular and serological assays, including RT-PCR, ELISA, and LAMP, highlighting sample sources, detection windows, and performance. Diagnostic considerations across human and animal hosts are discussed, with emphasis on outbreak applicability and field-readiness, given the need for diagnostic assays that are suitable for use in low-income areas. Further development of diagnostic assays, including isothermal amplification tests and other next-generation approaches, is recommended to fill the gap in rapid, point-of-care diagnostics.

## 1. Introduction

Hendra Virus (HeV) and Nipah Virus (NiV) are two members of the genus Henipavirus within the Paramyxoviridae family. Both viruses cause severe infection with high mortality rates in humans as well as in some domestic mammals. Found globally, fruit bats (Pteropodid bats) are the reservoir and have been implicated in initiating outbreaks. NiV, first identified in Malaysia in 1998, is primarily found in South and Southeast Asia [1]. HeV first emerged in 1994 in Brisbane, Australia, and all cases appear to be limited to Australia at this time [2].

A key constraint in work involving NiV and HeV is the fact that it must be conducted within BSL-4 laboratories. This severely limits the number of available laboratories and scientists that can work with the pathogens, as well as their proximity to cases and frontline healthcare workers. This further restricts research on new diagnostic assays, vaccines, and antiviral drugs, which is why the use of recombinant viruses has become a common strategy for circumventing this. However, this can also potentiate outbreaks when there are increased turnaround times or delays with diagnostic tests, that lead to the further spread of the virus to susceptible populations. Thus, effective and rapid diagnostic tests would improve the response time to disease outbreaks and thereby enhance mitigation strategies. Our goal is to provide a comprehensive overview of the current diagnostic platforms that are available for HeV and NiV while highlighting the advantages and disadvantages of each technique.

## 2. Hendra Virus

### 2.1. Historical Background of HeV

Hendra virus (HeV) is a zoonotic virus that first emerged in 1994, causing an outbreak of a fatal respiratory disease that affected twenty-one horses, of which fourteen died, and two humans in the Brisbane suburb of Hendra, Australia. The clinical signs in the affected horses included fever, anorexia, depression, ataxia, tachycardia, tachypnea, and nasal discharge. The two human cases presented influenza-like symptoms after reported direct contact with the sick horses. While one individual recovered six weeks later, the other succumbed after seven days of severe illness [3]. The virus was isolated and characterized from the lung and spleen homogenates of the deceased horses and the kidneys of the deceased human [4]. Four experimentally infected healthy horses reproduced similar respiratory symptoms and confirmed viral transmission, as the virus was successfully reisolated from the infected horses. Additionally, cytopathic effects with syncytia were observed in cell culture systems for several days post-inoculation, and neutralizing antibodies against the virus were found in the serum of both the horses and the humans who were infected [4]. These studies, along with genetic analysis, led to the initial identification of the causative pathogen of the outbreak as equine morbillivirus [4]. However, several comparative studies of its structural proteins revealed variations when compared to the sequences of other existing members of the Paramyxovirinae subfamily, which, along with its relatively large genome size (18,234 nucleotides), resulted in the creation of a new genus within the Paramyxoviridae family called Henipavirus [5,6,7,8,9,10].

### 2.2. Virus Structure and Classification

The equine morbillivirus was later renamed Hendra virus. It is an enveloped, non-segmented, negative-sense, single-stranded RNA virus with a pleomorphic shape (either spherical or filamentous). The size of HeV ranges approximately from 40 to 600 nm, and its surface is predominantly covered with projections of varying lengths that give it a double-fringed appearance [11]. Its genome encodes six major structural proteins, including N (nucleocapsid), P (phosphoprotein), M (matrix), F (fusion), G (glycoprotein), and L (polymerase) in the 3′–5′ order [12]. The viral envelope is predominantly covered with two glycoproteins, including the G protein that enables the virus to attach to the sialic acid-containing cell receptor, inducing a conformational change that allows the F protein to facilitate membrane fusion and viral entry [13]. In the ratio of 1 N per 6 ribonucleotides, the N protein binds to the genomic RNA, forming a helical nucleoprotein complex that serves as a template for transcription and replication. The P protein acts as a chaperone of the newly synthesized N protein and as a cofactor for RNA-dependent RNA polymerase L protein that supports viral replication and catalyzes mRNA transcription. In contrast, the M protein plays a vital role in viral assembly and budding from the host cell [12]. In addition to these structural proteins, three non-structural proteins (V, W, and C) encoded in the phosphoprotein are also part of the structure of the virus, which helps evade the host immune response by interfering with interferon signaling pathways [12]. Several factors of HeV, including a well-organized genetic constitution and a wide host range, contribute to a high pathogenicity and its ability to replicate efficiently in both animals and humans [14]. The capacity of HeV to form syncytia further emphasizes its potential to spread among cells. A few studies demonstrate that HeV is more unstable outside the host at higher temperatures than at lower temperatures and is sensitive to low pH, desiccation, and common disinfectants such as sodium hypochlorite and ethanol [15,16]. Nonetheless, HeV remains stable in saliva and urine from infected bats for short periods, which facilitates transmissions to animals and humans in the environment under ideal conditions [15,16].

### 2.3. Hosts and Reservoirs

Following the two initial outbreaks in Brisbane and Mackay, no serological evidence was found in the serum of the horse population collected during a survey study in those regions, suggesting the presence of a possible reservoir. Subsequent serologic sampling conducted on bats showed the presence of antibodies against equine morbillivirus in these species [17]. Further studies supported the hypothesis, and the primary reservoir of the HeV was later confirmed to be the Pteropus bat, commonly known as the flying fox [18,19,20,21]. These bats are asymptomatic carriers and shed the virus at any time of the year through their urine, feces, and saliva. Though seasonal reductions in food availability for flying foxes, especially from flowering trees, have been associated with an increase in HeV spillover events. Thus, hypotheses have been raised suggesting that climatic patterns such as El Niño and changes in eucalypt phenology influence bat foraging behavior and outbreak occurrence [22].

### 2.4. Transmission and Prevention

The virus typically spreads to horses through contamination of feed or water sources with infected bat excreta. Transmission to humans occurs primarily through close contact with infected horses, particularly during veterinary procedures or handling of bodily fluids from sick animals [23].

There have been no confirmed cases of direct flying fox–human or human–human transmission. The human infection rate is estimated to be 10% and has resulted from close contact with infected horses [24]. While horizontal transmission is the main way the virus spreads to horses, vertical transmission and the reactivation of latent infections have been observed in flying foxes. Transmission via the oral route was also reported to be possible [20]. Transmission between horses is inefficient and requires direct contact with infected bodily fluids. Other species, such as dogs, have also been found to be infected with HeV. For instance, a dog tested positive for HeV on a property in Queensland, Australia, where confirmed infected horse cases were identified [25]. To investigate the potential for cross-species transmission, experimental infection studies were conducted in cats and guinea pigs, which were found to be susceptible to HeV, showing signs of disease such as inappetence, respiratory distress, and pneumonia [21,26]. The prevention of HeV relies heavily on minimizing contact between horses and flying foxes and reducing the risk of horse-to-human transmission. There is currently no vaccine to prevent HeV infection in humans. However, a vaccine for horses, Equivac, has been available since 2012 [27].

### 2.5. Clinical Signs

Hendra virus infection in horses and humans can be severe and often fatal. In humans, the disease manifests after an estimated incubation period of 5 to 21 days with influenza-like symptoms that can easily progress to encephalitis, multiorgan failure, and fatality [28].

In horses, the incubation period is 3 to 16 days, a period after which HeV has been reported to cause neurological symptoms, including ataxia, depression, blindness, disorientation, head tilt, recumbency, facial nerve paralysis, seizures, and rapid deterioration, leading to death within a few days [29,30,31]. Infected horses can also exhibit respiratory symptoms such as frothy nasal discharge, fever, respiratory distress, and facial swelling [32]. The fatality rate is approximately 60% in humans and 75% in horses [33].

## 3. Nipah Virus

### 3.1. Historical Background of NiV

As mentioned elsewhere, the first reported human cases of NiV occurred in Malaysia in 1998 through direct contact with infected pigs [34]. Following this, there was another NiV outbreak in humans in 2001 in Bangladesh, and since then, there have been numerous outbreaks that occurred almost annually, usually with very high mortality rates. Following the first outbreak in Malaysia, cases were carried over into Singapore in 1999 [35].

### 3.2. Hosts and Reservoirs

**Figure A1:**
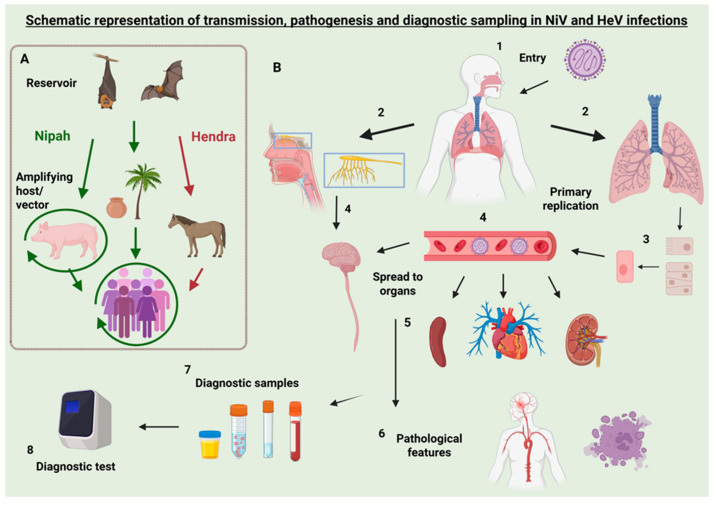
Transmission pathways, pathogenesis, and diagnostic sampling for Nipah and Hendra virus infections. Left panel (**A**) illustrates the transmission dynamics of Nipah virus (NiV) and Hendra virus (HeV). Both viruses are maintained in Pteropus fruit bats, the natural reservoir. Virus is shed in bat urine, saliva, and feces, facilitating environmental contamination and spillover. In the case of NiV (green), transmission to humans can occur either directly through consumption of contaminated date palm sap or indirectly via amplifying hosts such as pigs, with human exposure occurring through close contact. NiV may also spread between pigs and, less commonly, to other animals such as dogs. HeV transmission to humans occurs via infected horses, which act as intermediate hosts after environmental exposure to bat secretions. Horse-to-horse transmission of HeV is possible but less common. Right panel (**B**) outlines the pathogenesis and diagnostic workflow (steps 1–8) in human infection of NiV. 1: NiV virus uptake typically through oral mucosa or respiratory tract. 2: The virus typically enters the respiratory tract (lungs), but may enter the olfactory nerve. 3: Primary replication of the virus occurs at the entry site (typically the respiratory epithelium), and the virus subsequently enters endothelial cells. 4: The virus spreads to other organs through viraemia or directly to the rest of the CNS from the olfactory nerve. 5: Key target organs include the lungs, CNS, spleen, heart, and kidneys. 6: Pathological features include vasculitis, encephalitis, necrosis, and syncytia formation. 7: Diagnostic sampling may involve nasal or throat swabs, blood, urine, CSF, feces, or tissue samples. 8: Laboratory diagnosis using molecular or serological methods (e.g., PCR, ELISA, LAMP) is performed based on sample type and clinical presentation. Note: While the pathogenesis of HeV follows a similar pattern, some routes (e.g., olfactory nerve spread) are more conclusively demonstrated in NiV models. While this schematic primarily illustrates human infection with NiV, many pathogenesis features—such as mucosal entry, respiratory replication, systemic spread, and endothelial tropism—are shared with infections in animal hosts such as pigs and horses. Created in BioRender. van den Hurk, S. (2025) https://BioRender.com/pmk9s2s (accessed on 13 June 2025).

Nipah virus spread occurs primarily through animal reservoirs (bats) to intermediate hosts (pigs). While other animal species are affected, bats and pigs serve as the major species of concern for transmission and outbreaks [1,36]. Fruit bats of the genus *Pteropus* are the natural hosts of the virus and carry the virus asymptomatically, which allows the virus to be transported across vast regions. Through seroprevalence and experimental studies, it has been seen that that NiV can infect and be detected in multiple other domestic animal species including dogs, horses, cats, cattle, goats, and other animal species; while the association is not clear, some of these might have even been associated with human cases [37,38]. NiV may also spread between pigs and, less commonly, to other animals such as dogs. See Figure A1 for a schematic outline of the transmission cycle and reservoirs.

### 3.3. Geographic Distribution and Human Outbreaks

The two countries most highly affected by NiV outbreaks are Malaysia and Bangladesh. However, human outbreaks have occurred in three other countries, including Singapore, India, and the Philippines [35]. Different regions in India have been affected by many outbreaks of human cases, with very high mortality rates, which have occurred frequently since 2001 [39,40]. The first outbreak was reported in the Philippines in 2014, which was associated with horse consumption and contact, and the viral strain most closely resembled the NiV-M genotype [35]. Surveillance studies have shown that the virus is circulating in fruit bats in Cambodia, with multiple viral genetic clusters representing different sublineages of the virus [35]. Surveillance studies have demonstrated bats positive for NiV (asymptomatic reservoirs) in African countries, including Ghana and Madagascar, thus highlighting the risk of potential outbreaks beyond the current geographic regions [41].

### 3.4. Molecular Virology

Nipah virus is a negative-sense, single-stranded RNA virus that is approximately 18.2 k nucleotides in length [34]. The virus is pleomorphic, enveloped, and has a non-segmented genome [34]. The viral genome consists of six genes, with their six respective proteins being produced, and makes use of both mRNA editing and an alternative start codon to produce three additional proteins [37]. For diagnostic assay design, a concise understanding of the viral proteins is useful. Briefly, as a negative-sense RNA virus, NiV must encode its transcriptional complex, the viral ribonucleoprotein, which is activated upon the entrance of the host cell to facilitate further replication steps. For this purpose, the virus encodes N (nucleocapsid), P (phosphoprotein), and L (long polymerase) proteins, and the genes for these proteins are attached to the viral RNA, thus forming the ribonucleoprotein [37]. The M gene encodes the matrix protein, which helps determine the inner structure, while the F and G genes encode the fusion glycoprotein and attachment glycoprotein, respectively [36,37]. The attachment glycoprotein (G) binds to the cellular receptors, ephrin B2/3, which then induces conformational changes, affecting the F protein [42]. The fusion protein (F) codes for five potential N-glycosylation sites and is cleaved (broke into segments by enzymatic activity) into F1 and F2 subunits by the host protease.

The F1 subunit is implicated in the fusion of the virus and cell membranes after the induced conformational change from binding the receptor-binding protein G [12,37,42,43]. The F2 subunit has been shown to have a conserved region that is also important for fusion regulation in paramyxoviruses [44]. However, Moll et al. (2004) determined that the removal of the two glycoproteins (g2 and g3) from the F2 subunit had little–no effect on surface transport or fusion activity [45]. At the same time, the removal of g4 and g5 from the F1 subunit had a pronounced effect on surface expression and cell-to-cell fusion [45]. The virus uses the receptor binding protein, G, to attach to ephrin B2/B3 receptors, which then triggers the changes in the F proteins to mediate membrane fusion and viral entry into the host cell [43].

Two of the remaining proteins, the V and W proteins, are produced as products of the P gene through mRNA editing [37]. These two proteins inhibit Interferon production and signaling in mammalian cells and are thus important factors in pathogenesis and immune evasion [36]. The C protein, which is involved in the budding and release of viruses from the cytoplasm, is the last product of the P gene, and an alternative start codon is used for its production [36,37]. Altogether, this amounts to six structural proteins (N, P, M, F, G, and L) and three accessory proteins (V, W, and C) [39].

It is well recorded that there are two main distinct genetic lineages (genotypes) of NiV, NiV-M (Malaysia), and NiV-B (Bangladesh), which differ in genome length by 6 nucleotides (NiV-B is longer) [34,46,47,48]. Additionally, these two genotypes reportedly share about 91.8% nucleotide homology, with the differences being found throughout the genome [46]. Recent studies have also indicated that there might be four sublineages (minor genotypes) and fifteen genetic clusters of NiV associated with different regions, although with substantial overlap of these sublineages and clusters (cocirculation) in some regions. Cortes-Azuero et al. reported a clear geographic distribution in the circulation of these viral sublineages with differences in the proportions and in the identities of the viruses between Western and Eastern regions, namely, India, Bangladesh, Thailand, Cambodia, Indonesia, and Malaysia [35]. Within what was reported by Cortes-Azuero et al., the two main genotypes do contain what would be the NiV-M and NiV-B genotypes but do not refer to them directly (NiV-B is within genotype I, and M is within genotype II) [35]. Rahman et al. have further demonstrated sublineages in NiV-B based on the N gene, wherein NiV-B1 and NiV-B2 were identified [49].

### 3.5. Clinical Signs, Pathology, and Animal Models

Nipah virus causes a severe and commonly fatal disease in humans and in some animals. Multiple studies have tried to evaluate the differences between different genotypes of NiV, and to determine whether these might be related to differences in the geographic distribution, transmission, and the clinical picture seen in humans [50,51,52,53]. However, there appear to be differences in findings between studies, as well as variability between animal models used to study NiV-B and NiV-M. The animal models most commonly used for NiV studies are hamsters, ferrets, and African green monkeys. Hamster models have been considered to be the best rodent model for recapturing the elements of human NiV infection, especially neurological changes and the pathology of the central nervous system (CNS) [50,51]. Some studies have reported that NiV-M strains lead to accelerated virus replication, more severe disease pathology, and faster death than NiV-B, particularly in a Syrian hamster model and in cell culture [50]. Other studies have suggested that the NiV-B genotype may be more pathogenic and that it exhibits increased oral shedding compared to NiV-M, as observed in African green monkeys and ferrets, respectively [34,52]. These animal model observations align with outbreak findings, where NiV-B has been associated with enhanced respiratory symptoms and transmissibility. Aerosolized and intranasal exposure models using NiV-B (advancing on the intratracheal route) were reported to more accurately reflect natural mucosal exposure and human disease progression [48,54]. African green monkey experimental models are cited as being the most consistent model to fully capture all elements of human NiV disease, including generalized vasculitis as well as respiratory and neurological disease in human cases [53].

Griffen et al. (2019) reported the development of a reverse genetics system for NiV-B and NiV-M strains, which could generate infectious cDNA clones that seemed to perform well in vitro and in a Syrian hamster model [55]. This model should be helpful in the development of potential drug and vaccine candidates. Researchers demonstrated that NiV-B could be used to infect a swine model by infecting pigs via oronasal inoculation; however, unlike with NiV-M infection, clinical signs and viremia were not found in infected pigs [56]. Additionally, only low levels of circulating antibodies were found in these infected pigs. These are important findings when considering surveillance and control measures that might rely upon serological surveys, and further studies should evaluate if pigs might have a role in the circulation and transmission of NiV in Bangladesh that has not previously been described.

Beyond considering these different virus strains, researchers have noted that it is important to consider that some of the differences observed in disease manifestations and outbreaks might be related to a combination of the route of transmission, as well as the social, cultural, and environmental factors that would affect virus transmission [57]. This is supported by the aforementioned studies, where the inoculation of the virus into the respiratory system and as an aerosol was able to reproduce the same respiratory clinical signs as found in some NiV-B infections, with less emphasis on the encephalitis symptoms.

Observations from previous outbreaks suggest that human cases of NiV-B were derived from infected bats through exposure to contaminated food, while NiV-M cases were largely derived through close contact with infected pigs [56]. In addition to the transmission of cases from animal hosts, other differences are noted through comparison between human outbreaks of NiV-B and NiV-M. Records from outbreaks indicate that NiV-B has a higher case fatality rate of 70–100%, compared to a case fatality rate of around 40% for NiV-M outbreaks [56]. Additionally, respiratory disease and shedding seem to be a bigger component of NiV-B compared to NiV-M cases, and this has been noted as a likely contributing factor to disease transmission between humans in NiV-B cases [47,56]. Human-to-human spread is a major mechanism of transmission in NiV-B, which is different in NiV-M, where past outbreaks have typically not demonstrated transmission between humans, but rather only from pigs to humans [47].

The cases that have occurred in Bangladesh demonstrated human-to-human transmission, although they were found to have emerged from the consumption of food products (date palm sap) contaminated mainly by the bodily fluids of fruit bats [34]. Pigs may act as amplifying intermediate hosts and transmit the virus to humans, as seen in previous outbreaks with NiV-M [37,56]. The initial Malaysian outbreak demonstrated that vocations or other activities leading to direct contact and exposure with pigs are risk factors for infection [38]. The outbreak in Bangladesh demonstrated that consumption of contaminated date palm sap led to infection; thus, the practice of consuming date palm sap would increase the risk of virus transmission [58]. Correspondingly, keeping pigs in an environment where fruit bats might consume and shed the virus could increase the risk of pigs being exposed to NiV. Potential vocation risks of NiV transmission to healthcare workers are associated with exposure to an infected person before a positive diagnosis is made and before appropriate precautions are implemented.

Some of the pathological findings include systemic vasculitis and parenchymal necrosis, which is characterized by endothelial cell damage, necrosis, and syncytial cell formation within affected vessels and organs [59]. The formation of thrombi, microinfarcts, vasculitis, and the infiltration of inflammatory cells is common, and all of these pathological changes may be found in the major organs, and typically involve the central nervous system, lung, kidneys, spleen, and heart [41,60]. Animal models suggest that the route of infection/transmission might have an impact on the sequence of pathological changes and the clinical syndrome that is expressed. However, the described vasculitis and endothelial cell necrosis are present with all infections and may lead to systemic disease and death when this affects different organ systems. There might be increased respiratory pathology and involvement in cases where virus inoculation occurs within the respiratory tract, or in the late stages of infection; in both cases, broncho-interstitial pneumonia may be seen, as well as an inflammation-mediated (cytokine/chemokine-induced) respiratory pathology and distress [41,59,61,62]. As previously described, infection with NiV-B also seems to result in increased respiratory disease compared to NiV-M strains.

A proposed pathway for the pathogenesis is that the virus may enter through the mucus membranes of the mouth or nose, whereafter it can either enter the respiratory tract or the olfactory nerve [63]. If it enters the olfactory nerve, the virus will enter the CNS to cause encephalitis and other neurological diseases resulting from the mechanisms described. If it enters the respiratory tract, the virus will be transported from epithelial cells to enter the endothelial cells, where there will be syncytia formation and the vasculitis and other pathologies. Once the virus is in the endothelial cells and vasculature, it is dispersed across the body to the other organ systems and will likely enter the blood–brain barrier and cause CNS pathology [59,63,64]. Interestingly, the virus does not seem to cause pathological lesions in the liver or skeletal muscles of humans [63]. However, experimental studies in animals have demonstrated hepatic congestion and hepatic sinusoidal leukocytosis [48]. An additional pathological finding in animal models is hemorrhage and congestion in the urinary bladder, which is fitting considering that the virus is known to be shed in the urine of infected animals (including reservoir and subclinical animals) [37].

The clinical picture in humans infected with the NiV is variable, but there is typically severe disease and very high mortality rates. Early stages of infection may typically involve non-specific symptoms of viral infection, such as fever, headache, malaise, myalgia, dizziness, and nausea [59,64]. Patients typically worsen with progressing disease, and symptoms may include encephalitis and other neurological diseases, and/or respiratory disease symptoms such as severe pneumonia (and associated symptoms) [59]. The incubation period varies from a few days to two months, with an average of 5–7 days [41]. Neurological disease symptoms associated with encephalitis (acute onset and delayed onset) are common, and the symptoms are very variable but may include seizures, focal neurological deficits, seizures, and coma. Patients might recover, succumb to infection, or experience relapsing episodes of encephalitis [41,65]. Neurological disease symptoms appear to be more common with NiV-M infection, as opposed to NiV-B, although fatality rates with NiV-M infection are typically lower.

## 4. Diagnostic Methods

Making an accurate and rapid diagnosis of HeV and NiV infection depends on clinical awareness and laboratory identification of viral antigens or antibodies. Early diagnosis, supported by molecular and serological tests, is critical for improving outcomes, controlling outbreaks, and managing animal movements. Table A2 and Table A3 provide summaries of some of the different diagnostic assays used for detection of HeV and NiV.

### 4.1. Molecular Tests

#### 4.1.1. Nucleic Acid Amplification Tests

Currently, the gold standard for diagnosis of HeV and NiV is reverse-transcription quantitative polymerase chain reaction (RT-qPCR), and this assay is highly sensitive and specific and is the method recommended by the World Organization for Animal Health (WOAH) [66]. Garbuglia et al. (2023) [67] provided a thorough review of diagnostics for NiV and included an excellent table outlining different molecular diagnostic assays; we recommend that the reader refers to this [67]. The most common target for RT-PCR is the N gene of NiV and HeV, but some tests have also targeted the L, M, and P genes, the matrix (M), and the F- and G-protein-coding regions [67,68,69]. These assays offer very impressive sensitivity, with a limit of detection (LOD) as low as 10–100 copies/reaction and 20 copies/reaction from tests using SYBR Green, and another with a fluorescent reporter dye [67].

Considering that many HeV and NiV outbreaks have occurred in resource-limited regions, it is valuable to have diagnostic assays that are low-cost, do not require specialized equipment, and are ideally suitable for point-of-care (POC) testing. Chen et al. [70] developed a POC assay that made use of a one-step RT-PCR, lateral flow immunoassay, and microfluidic technologies, which achieved an LOD of 199.1 copies/reaction [70]. Their assay targeted the G and P genes, and achieved high specificity in their tests while also enabling the simultaneous detection of NiV-B and NiV-M strains [70]. A low-resource rapid test based on reverse-transcription isothermal recombinase polymerase amplification (RT-RPA) has also been developed to detect HeV [71]. Isothermal amplification assays are well suited for addressing concerns regarding the available resources by reducing the need for thermocyclers and other specialized equipment while providing a reliable assay with high sensitivity and specificity. The two main isothermal amplification assays that have successfully been used to develop diagnostic assays for HeV and NiV are RT loop-mediated isothermal amplification (RT-LAMP) and recombinase polymerase amplification (RPA) assays. A highly sensitive RT-LAMP assay was developed to detect HeV before the onset of clinical signs [72]. Ma et al. (2019) developed an RT-LAMP assay targeting the highly conserved region of the N gene of NiV [73]. The assay had a very high sensitivity with an LOD of (110 pg of total virus RNA), and high specificity, with no cross-reactions with the N gene protein from other viruses tested [73]. Isothermal amplification assays were tested further by Pollak et al. (2022), who evaluated two RPA assays and a recombinase-aided amplification (RAA) assay for NiV [74]. Their assays also targeted the N gene, and all three assays demonstrated 100% specificity in their tests. The best-performing assay (an RPA assay) had a 100% sensitivity and an LOD of 1000 copies/μL, while the worst-performing assay, the RAA assay, had a sensitivity of 62.5% [74]. Above what has been mentioned, these RPA assays are promising for field use as the protocol included an inactivation step (making it safe for further processing), was effective on the NiV-B and NiV-M strains. Additionally, the whole testing procedure took 30 min from the start of sample preparation to reading the result [74]. Miao et al. (2023) developed an RPA-CRISPR/Cas13a assay which was a one-pot assay for NiV and had an LOD of 1000 copies/μL of total synthetic cDNA [75]. The assay had high specificity with no cross-reactions with other viruses tested, and the results could be visualized with fluorescence or lateral flow strips [75]. One limitation that is encountered with most of the studies and molecular assays referenced is that the tests were conducted on pseudoviruses, spiked samples, or other artificial samples only; this is understandable and practical, but it is possible that the results might therefore not fully reflect those that can be expected from actual samples from patients with natural NiV infection.

#### 4.1.2. Sequencing

Sequencing techniques have been used for research and epidemiological studies for some time [39]. Although Sanger sequencing and next-generation sequencing (NGS) techniques do not appear to have been used for the clinical diagnosis of HeV and NiV yet, this is something that should be considered. Sequencing technology has evolved and improved rapidly in recent times, alongside a dramatic reduction in costs, and this increasingly justifies the use of this technology for outbreak detection and diagnostics. Bonsall et al. (2020) published an example of how a high-throughput, cost-effective NGS workflow and computational pipeline had been developed for surveillance and clinical monitoring of HIV in low-income settings in Zambia [76]. The researchers reported that their method was able to generate full genomic sequences of diverse HIV strains, enabled drug resistance surveillance, and provided valuable information to construct directed transmission networks [76]. A similar approach and technology for HeV, NiV, and other emerging viruses would be highly valuable in resource-limited settings to aid accurate diagnostics and enhanced transmission tracing.

### 4.2. Serological Tests

#### 4.2.1. Immunosorbent Assays

Multiple different enzyme-linked immunosorbent assays (ELISAs) have been developed and tested for NiV and HeV; these can broadly be categorized as antigen detection ELISAs and antibody detection ELISAs. ELISAs can further be classified based on their mechanism or the type of test, including direct, indirect, competitive, and sandwich ELISAs, each with their own variations and specific applications [77]. While direct ELISAs are not commonly utilized for NiV or HeV diagnostics, numerous indirect, competitive, and sandwich ELISAs have been specifically developed and refined for detecting viral antigens or specific antibodies to these viruses. Table A2 evaluates some of these different assays as evaluated for HeV and NiV. Many of these ELISAs utilize tetramethylbenzidine (TMB) as a chromogenic substrate, which allows for convenient reading of results through a clear color change [78].

Serological diagnostic assays targeting antibodies should be carefully considered to ensure they align well with diagnostic objectives, such as confirming active infections, past exposure, or evaluating immunity status. Broadly, antibody detection assays target immunoglobulin M (IgM) and immunoglobulin G (IgG). Detection of IgM antibodies represents acute infection, and for NiV infection, research indicates that these might be present from the onset of symptoms (or 3–5 days following disease onset) until around day 27 of infection [79]. Some researchers suggest that these IgM antibodies may even be detected as late as 3 months, and while less typical, even up to 7 months following the onset of disease [67,80]. IgG antibodies, indicative of past exposure or long-term immunity, typically become detectable around day 17 and may persist from eight months to over one year post-infection [67,79,80].

The broad and variable detection windows highlight one of the potential limitations of serological tests, namely their dependency on host antibody production. A diminished antibody response in certain hosts can negatively affect test results. Additionally, the timing of sample collection is crucial; samples collected too early or too late could lead to false-negative results.

Therefore, the selection of an appropriate ELISA depends on the specific diagnostic scenario, desired sensitivity and specificity, and feasibility of batch testing. Table A2 in the Appendix A provides a summary of some of the different ELISAs used and investigated with NiV and HeV. It is also essential to evaluate whether an ELISA is the most suitable diagnostic test compared to molecular methods such as PCR, RPA, or LAMP, particularly for acute phase diagnosis or when rapid, highly sensitive detection is required. Serological tests to detect HeV were initially developed at the CSIRO Australian Animal Health Laboratory. Indirect ELISAs are most commonly employed for antibody detection, with several assays demonstrating good specificity and reliability for both NiV and HeV.

We evaluated three studies presenting indirect ELISAs that were developed for NiV and HeV antibody detection in pigs and humans [78,81,82]. All three of these studies used recombinant NiV proteins as the target proteins, and all three used *E. coli* as the expression system to produce NiV nucleocapsid protein, and affinity chromatography for purification. For all the indirect ELISAs, the recombinant NiV protein was used to coat the plate and target the sample antibodies before the washing and addition of the enzyme conjugate [78,81,82]. The use of recombinant target proteins for NiV is an effective approach for bypassing the necessity for high-level biosecurity laboratories and the safety concerns associated with the complete and wild-type virus.

In the first study, Yu et al. (2006) developed an indirect ELISA for the detection of IgG antibodies in humans and swine, and a capture ELISA for IgM antibodies in humans (antibodies were captured with anti-human IgM antibody) [81]. The indirect IgG ELISA performed well (good sensitivity and specificity) for pigs and humans when compared to a CDC-developed (Centers for Disease Control and Prevention) ELISA, although sample numbers, particularly pig samples, were low in the study. The IgG assay performed better than their IgM capture ELISA, although their IgM ELISA detected more positives than the CDC IgM ELISA, particularly pig samples [81]. Kulkarni et al. (2016) developed an indirect ELISA targeting IgG antibodies to NiV-N protein in pigs (no human samples tested), and tested 1709 swine serum samples from across India [78]. The assay performed well with high sensitivity and specificity, and the assay made use of tetramethylbenzidine (TMB) as a chromogenic substrate to facilitate reading by color change [78]. These factors support the use of the assay for large sample numbers and screening. Fischer et al. (2018) developed three indirect ELISAs for detecting Henipavirus-specific IgG antibodies in pigs, with differentiation between HeV and NiV [82].

The initial screening ELISA used the recombinant NiV N protein, which was broadly reactive for both HeV and NiV. The two other ELISAs used specific glycoproteins, either recombinant HeV-G or NiV-G, for HeV or NiV, respectively, in order to differentiate and confirm the respective virus. The HeV-G and NiV-G proteins were expressed by *Leishmania tarentolae* to ensure eukaryote (mammalian-like) glycosylation to ensure better antigenicity [82]. These assays also made use of TMB to facilitate reading by color change and optical density as an objective measure, facilitating its use as a high-throughput screening assay for NiV and HeV in pigs.

An antigen-capture ELISA, as found in a sandwich ELISA, can be used for detecting active infections from patient samples. Yang et al. (2022) developed a sandwich ELISA utilizing recombinant human ephrin B2 protein as a capture ligand for NiV and HeV antigens [83]. The assay successfully differentiated between NiV and HeV using monoclonal antibodies (one for NiV and one that cross-reacted with NiV and HeV) and enabled the estimation of viral loads based on detected antigen concentration. The researchers also developed a lateral flow strip test for NiV and HeV antigens using biotinylated recombinant ephrin B2 and the same monoclonal antibodies. The lateral flow gave rapid results in 15–30 min, based on ephrin B2 as a universal ligand, and well suited to field use; however, it had lower sensitivity than RT-PCR and was limited in its detection of HeV [83].

Competitive ELISAs can be used for both antibody and antigen detection, depending on the design of the assay. Researchers developed a competitive ELISA to detect antibodies to NiV and HeV by making use of recombinant Nipah glycoproteins and monoclonal antibodies [84]. The assay used recombinant NiV glycoprotein G (NiV-G), to which NiV and HeV antibodies cross-react, to coat the plate. The competing monoclonal antibodies (mAb) specific to the NiV-G either bind or are inhibited by sample antibodies (inhibition indicates a positive result) [84]. The assay offered high sensitivity and specificity for NiV and HeV antibody detection and could be used across multiple animal species (pigs, mini pigs, and non-human primates were tested) without necessitating species-specific antibodies or reagent changes [84]. An additional benefit of this competitive ELISA is that, by using recombinant virus glycoprotein, it eliminates the need to handle live virus, thereby removing the requirement for BSL-4 laboratory facilities to use the assay. These characteristics mean that the assay is a particularly well-suited screening test for antibodies in both animal and human populations, especially in remote or resource-limited settings.

Sheete et al. (2022) developed a capture ELISA for anti-NiV IgM antibodies in human serum, and an indirect ELISA for anti-NiV IgG antibodies [79]. These ELISAs demonstrated very high sensitivity and specificity rates on the samples tested, including human serum samples from a previous outbreak. The study highlighted some of the main benefits of ELISA assays in that they were cited as being cost-effective, did not require extensive equipment, and thus were well suited to large-scale epidemiological screening [79].

A solid-phase blocking ELISA (SPB-ELISA) is a specialized type of competitive ELISA that can be used for antigen or antibody detection and is highly specific due to reduced cross-reactivity in the assay. Kashiwazaki et al. (2004) developed a solid-phase blocking ELISA making use of monoclonal antibodies specific to NiV glycoprotein to detect NiV-neutralizing antibodies in multiple animal species [85]. An SPB-ELISA is well-suited to surveillance and screening studies and attaining a high throughput of tests. The researchers tested swine, equine, bat, and human samples, and achieved moderate sensitivity and high specificity [85]. This approach is said to be simple and does not require species-specific reagents, which makes it easier to use it in testing multiple animal species. These factors could make this assay helpful for outbreak surveillance and population screening, which can then be followed up with a more sensitive assay [85].

A Luciferase Immunosorbent Assay (LISA) was developed by Li et al. (2024) as an alternative to ELISA for the detection of NiV-G protein IgG antibodies [86]. The researchers made use of a pNLF1-N expression vector to express recombinant NiV-G protein as a luciferase fusion antigen for the assay. For this assay, the plate was first coated with protein G (an immunoglobulin-binding protein) to capture IgG antibodies (not species-specific) from the serum, which then bind the recombinant NanoLuc-labeled NiV-G fusion protein. A reaction occurs when the NanoLuc substrate is added, which results in fluorescence measured as relative fluorescence intensity (RFI), as opposed to a colorimetric change, which necessitates a fluorescence reader [86]. The direct ELISA had good specificity on cross-reaction studies and was four times more sensitive than the indirect ELISA performed when comparing the limit of detection on NiV-positive mouse and horse serum. In the study, the authors tested multiple components of the NiV G protein for the assay, and they determined that the head domain within amino acids 176–602 was the optimal antigen fragment for IgG detection [86]. More studies need to be performed to test samples from humans and other animal species, as well as more field samples. However, this assay appears to be well suited for the large-scale testing and screening of multiple species with rapid and high-throughput processing. This could be preferable to SPB-ELISA for screening large numbers of samples from multiple species. Developments should also be made to allow for a field-suitable fluorescence reader to allow this to be used more widely and to be of greater help for surveillance.

A Luminex multiplex assay uses fluorescently labeled microspheres (beads) suspension arrays to detect multiple targets, meaning both multiplexing and the potential for the detection of antigens, antibodies, and nucleic acids. The Luminex platform enables the simultaneous detection and quantitation of multiple analytes in a single sample. This approach was used in two studies for NiV and HeV, one focusing on antigen detection and the other on antibody detection [87,88]. A significant advantage of the Luminex system over molecular assays like qPCR is that it allows for the detection of protein/gene targets simultaneously, such as targeting the nucleoprotein and phosphoprotein of both NiV and HeV (allowing up to 100 markers per reaction) [87]. In their study, Foord et al. (2013) found that the Luminex assay provided good sensitivity (improved for the N gene over the P gene) and specificity with an LOD similar to that of the qPCR assay when testing NiV and HeV isolates and HeV clinical samples [87]. The strong advantage of the system over PCR or sequencing assays is the ability to multiplex and to simultaneously evaluate multiple gene targets. However, there are limitations in that it still requires equipment and steps for nucleic acid amplification from the sample and also requires the Luminex system for evaluation and reading results. Thus, this diminishes its use in the field as a point-of-care test, but it could still be beneficial for reading large numbers of samples in a laboratory setting.

McNabb et al. (2014) used Luminex technology to test for antibodies against HeV glycoprotein (G) by making use of recombinant soluble glycoprotein (sG) from HeV and NiV (explaining why cross-reactions with NiV antibodies were seen) [88]. They performed a Luminex binding assay (binding of antibodies to sG), and a Luminex blocking assay (testing serum antibodies block of the binding of sG to the receptor ephrin B2). The binding assay was used as a comparison to an indirect ELISA, to which the Luminex assay performed better than the ELISA in terms of sensitivity and specificity, with less non-specific binding in their tests [88]. The blocking assay was performed as a surrogate for a virus neutralization test (VNT), as it is seen to be a faster and safer option, and it was cited to offer diagnostic value with a high specificity, although the sensitivity was reduced. The block assay had a lower range of detection compared to the binding assay, and analytical sensitivity approximately nine times lower than the virus neutralization test [88]. However, the Luminex assay was still useful for detecting neutralizing antibodies and correlated well with the VNT, making it a potentially safe and rapid option for screening large numbers of samples at a lower cost. Further developments in technology could make Luminex very helpful for testing large numbers of samples for multiple targets in outbreak situations.

#### 4.2.2. Neutralization Tests

The plaque reduction neutralization test (PRNT) is considered the gold standard for the serological confirmation of NiV and HeV antibody neutralization [67,89]. The PRNT is highly sensitive; however, it requires the use of live virus (necessitating a BSL-4 laboratory) and takes a number of days (3–7 days) to complete the assay. The virus neutralization test (VNT) is an alternative to PRNT, which may have slightly lower sensitivity while still being effective for antibody detection [67,89,90]. While VNTs are usually performed with replication-competent viruses, it is possible to perform tests using pseudovirus (pseudovirion-based neutralization assay), which allows the tests to be performed in BSL-2 or BSL-3 conditions instead [90].

#### 4.2.3. Immunohistochemistry

Immunohistochemistry (IHC) is primarily used to detect antigens in tissue samples from necropsies (usually formalin-fixed) with the use of antibody-based staining [89,90]. Immunohistochemistry is valuable for identifying the presence of viruses in different tissues and cells in order to understand pathology and pathogenesis, which would support post-mortem evaluations. However, PCR is likely better suited as a first-line diagnostic tool where the objective is to determine the presence or absence of the virus in tissue samples.

#### 4.2.4. Virus Isolation

Virus isolation is used to determine the presence of replication-competent viruses, and it was performed more commonly before the widespread availability and uptake of PCR to confirm HeV and NiV infection [18,89]. A virus is grown from submitted diagnostic samples on a cell line (such as Vero E6 cells) for multiple days and is evaluated for signs of cytopathic effect (CPE) [67,89]. Additionally, the propagated virus could be further evaluated using whole-genome sequencing or virus neutralization tests. This method is time-consuming to perform and not very sensitive, since not all diagnostic samples contain viable viruses that can be grown. Virus isolation must be carried out in BSL-4 laboratories and is not suitable for large sample volumes; both of these factors, coupled with advances in other methods, make it less desirable as a tool for laboratory diagnosis [91].

#### 4.2.5. Electron Microscopy

Electron microscopy (EM) is used to visualize virus particles for physical identification (to a family level) based on morphology and ultrastructural features [89,92]. This requires the use of specialized, large, and expensive equipment within laboratories with skilled personnel to run the test and for interpretation. Additionally, EM is labor-intensive, typically requires high virus concentrations in samples, and must be carried out in BSL-4 laboratories when using live NiV or HeV [89]. Electron microscopy was more widely used in the past and has largely been replaced by molecular techniques for routine diagnostic purposes of NiV or HeV, and is likely mainly used for research or investigation of novel agents in modern times [92,93].

## 5. Diagnostic Considerations for Prevention and Control

Although this review centers on diagnostics, an understanding of current control options helps frame assay requirements and provides deeper context for the application of the content.

There are currently no approved antiviral treatments or vaccines for NiV and HeV in humans or animals, although an animal vaccine for HeV exists. Potential vaccines and antiviral drugs are under active investigation, with multiple antiviral drugs being researched for their efficacy in inhibiting the replication and spread of NiV (see Appendix A.2 for further information).

Vaccination represents an important component of an ideal control strategy for NiV and HeV. In line with a One Health approach for addressing the risks posed by these Henipaviruses, the vaccination of humans and high-risk intermediate or reservoir host animal species would help to reduce disease transmission. Several potential vaccine candidates for humans and animals have been investigated, some of which are in development and further research trials (see Appendix A.2 for additional information).

A holistic approach to control potential outbreaks and prevent spillover events needs to focus on the different pathways of transmission and include surveillance and interventions at each level. Passive surveillance, while not a measure of control directly, can help to guide interventions, policy, and the allocation of resources. When selecting assays, the target population (e.g., infected human or reservoir bats) and purpose (e.g., diagnosis or surveillance) must be considered.

Molecular tests such as PCR remain the gold standard for the diagnosis of acute infection when infection is suspected. For these to be effective, healthcare providers must be adequately trained to suspect NiV and HeV infection and to request the correct diagnostic assay, which will help alert the healthcare system to a potential outbreak. However, passive antigen or antibody surveillance within high-risk animal populations (e.g., Malaysian pig farms in high-risk areas) with assays suited for large volume and population screening, such as capture ELISAs or Luminex assays, could be valuable.

This highlights the need for effective One Health interventions that involve the consideration of multiple factors simultaneously.

## 6. Conclusions

Hendra and Nipah viruses are zoonotic agents of significant public health importance, with the potential to spread as emerging pathogens in different parts of the world. At this time, cases of NiV and HeV have been limited to Asia and Australia, respectively. However, the habitat range of the animal hosts and vectors (Pteropodid bats) extends well beyond these regions, creating the potential for disease transmission events in Africa and other continents. Additionally, there are currently no vaccines available for human use for either of these highly fatal diseases. This highlights the importance of reliable diagnostic assays that are suited to resource-limited environments. This review highlighted the different diagnostic assays that have been developed for the detection of Henipaviruses and discussed the relative strengths and limitations of these assays. Assays that are widely used include RT-qPCR and ELISAs targeting N and G proteins. However, this approach presents challenges regarding DIVA (differentiating infected from vaccinated animals) with HeV, where horses may be vaccinated. Considering the epidemiology and disease dynamics of these viruses, certain assays may have more defined use cases (different assays were evaluated in the tables above). There is a need for assays that can be used for large-scale surveillance, assays suitable for point-of-care testing, and highly specific assays that can be used for outbreak confirmation. It is likely that molecular tests such as PCR-based assays will remain dominant for the direct detection of viruses or viral antigens. Currently, the availability of lab-based equipment (such as thermocyclers used in PCR reactions) does present a limitation to their widespread deployment. Diagnostic assays based on RPA or LAMP, which utilize isothermal amplification, may offer the potential for developing assays that can be transformed into point-of-care diagnostics, well-suited for field surveillance and testing in low-resource areas.

Looking forward, assays need to be assessed for their performance in field outbreaks and our ability to differentiate infected from vaccinated animals (and humans), which will be beneficial when vaccines become available. Lastly, there is little doubt that a One Health approach is essential when considering the control of these viruses. To achieve lasting success, we must ensure that our diagnostic capacity, vaccine development, disease surveillance, and passive control efforts are integrated across human and animal health sectors.

## Data Availability

No new data were created or analyzed in this study.

## Appendix A

### Appendix A.1

**Table A1:** Timeline of some of the major outbreaks, research milestones, and vaccine development for Hendra and Nipah viruses.

Year	Event	References
1994	Hendra virus first outbreak in Brisbane, Australia (horses and human deaths)	[3]
1995	HeV experimentally reproduced in horses; first human fatality	[4]
1995	HeV genome characterized; virus named equine morbilivirus	[4]
1998	NiV outbreak in Malaysia, linked to pigs	[34]
1999	NiV virus Singapore cases from imported pigs	[35]
2000	HeV isolated from Pteropus bats, confirming reservoir	[18]
2001	First NiV outbreak in Bangladesh and in India	[34,39]
2010	African green monkey NiV model established	[53]
2012	Equivac HeV vaccine released	[27]
2014	Philippines NiV outbreak linked to horse contact	[35]
2024	15 NiV genetic clusters reported by Cortes-Azuero et al.	[35]

**Table A2:** Comparison of some of the different immunosorbent assays that have been explored for use with Nipah and Hendra viruses. Note that the table is not comprehensive and does not include all studies, species, and targets of different assays.

	Indirect ELISA	Competitive ELISA	Sandwich ELISA	Capture ELISA	Solid-Phase Blocking ELISA	Luciferase Immunosorbent Assay (LISA)	Luminex Array Assay
Assay target	Antibody (IgG, IgM)	Antibody or Antigen	Antigen	Antigen and Antibody	Antigen and Antibody (neutralizing antibody)	Antigens, Antibodies	Antigen and antibodies (multiple targets)
Mechanism	Antigen (recombinant NiV) coats plate, sample antibodies bind, detection by enzyme-conjugated secondary antibody	Antigen (or antibody) coats plate, antibody (or antigen) competes with a labeled reference for binding, signal is inversely proportional to concentration	Captures antigen between two antibodies (capture and detection), typically using mAbs	Uses antibody or antigen for initial capture step, uses second antibody for detection similar to sandwich ELISA	Antibody binds to plate, blocks non-specific binding, detected via secondary antibody	Uses luciferase-based detection for enhanced signal	Bead-based multiplex technology with fluorescent detection
Species used	Tested in most species; studies included humans and pigs	Pig, mini pig, non-human primate	Human	Human	Swine, equine, bat, human	Mouse, horse	Human, horse
Potential use	Checking antibodies for infection, checking vaccination response/status, serological surveillance,	Broad species testing, detection of low-concentration samples	Active infection diagnosis, viral load estimation	Acute infection detection	Surveillance, high-throughput testing	Large-scale screening, high-throughput testing	Multiplex detection of multiple targets, outbreak screening
Advantages	High sensitivity, Recombinant NiV/HeV virus avoids BSL-4	High sensitivity and specificity	Can differentiate NiV and HeV, dose-dependent viral dose estimation	Good sensitivity and specificity, robust signal	High specificity, does not require species-specific reagents	Very high sensitivity, species-independent detection	Allows multiplex detection of multiple analytes
Disadvantages	Can achieve cross-reactions with secondary antibodies	Requires labeled competitor (monoclonal antibodies)	Lower sensitivity than RT-PCR, requires Mabs, requires high-affinity antibodies	More steps, antibodies determine results (may have reduced sensitivity)	Moderate sensitivity (confirm with second test), requires optimization for large-scale use	Requires specialized equipment, not readily available/tested in field conditions	Requires bead-based technology and flow cytometry, not suited to POCT/field use
Studies/References	[78,81,82]	[84]	[83]	[79]	[85]	[86]	[87,88]

**Table A3:** Comparison of some of the different diagnostic assays (excluding ELISA) that are commonly used and have been explored with Nipah and Hendra viruses. Note that the table is not comprehensive and does not include all studies, species, and targets of different assays.

Assay	Mechanism	Target(s)	Key Features	Use Case	Limitations/Notes	References
RT-qPCR	Reverse-transcription + quantitative PCR	N (main), L, M, P, F, G genes	Gold standard; WOAH-recommended; high sensitivity/specificity	Confirmatory diagnosis of acute infection	Requires thermocycler, RNA extraction	[66,67,68,69]
RT-RPA	Isothermal recombinase polymerase amplification	N gene	Field-suitable, inactivation step included, works for NiV-M and NiV-B	Rapid low-resource molecular testing	Sensitivity varies by assay (e.g., 100% to 62.5%)	[74]
RT-LAMP	Isothermal amplification (loop-mediated)	N gene	Highly sensitive; used for HeV preclinical detection	Surveillance or early diagnosis	Needs validation in field samples	[72,73]
RPA-CRISPR/Cas13a	One-pot isothermal and CRISPR detection	Synthetic cDNA of NiV	Visual detection via lateral flow/fluorescence; no cross-reactions	Lab-based rapid detection	Only tested on synthetic samples	[75]
PRNT/VNT	Live virus or pseudovirus-based neutralization	NiV and HeV neutralizing antibodies	Gold-standard serological confirmation	Confirming neutralizing Ab presence	Requires BSL-4 (live virus) or pseudovirus system	[67,89,90]
IHC	Antibody staining of virus antigen in tissue	NiV and HeV proteins	Used in formalin-fixed tissue; supports pathology studies	Post-mortem evaluation	Not first-line diagnostic	[89,90]
Virus Isolation	Virus grown in Vero E6 or other cell lines	Viable virus	Enables sequencing, further testing	Confirming infectious virus	Time-consuming, low sensitivity, BSL-4 required	[18,67,89,91]
Electron Microscopy	Visualizes virion ultrastructure	Virus particles	Historically used for genus-level ID	Primarily a research tool	Needs high virus concentration, BSL-4	[89,92,93]

### Appendix A.2. Current and Investigational Control Strategies for Henipaviruses (NiV/HeV)

Some of the antiviral drugs researched for HeV and NiV include common drugs such as ribavirin, remdesivir, and favipiravir, as well as less common potential drugs such as ALS-8112 and GS-5734 [94]. Some of these antivirals show promising results in vitro or in initial animal studies (or an early human outbreak for ribavirin). However, further development and testing of antiviral drugs are needed to achieve practical success where the onset of infection is unknown, and the delayed start of treatment is common. Monoclonal antibody treatments might provide a helpful alternative to antiviral drugs, especially when given in acute stages of infection. However, some treatments show potential efficacy even in later stages of infection in animal studies [94].

Some of the vaccines for NiV that are being investigated for use in humans include mRNA vaccines, a recombinant measles virus vaccine, a recombinant vesicular stomatitis virus vaccine, an adenovirus-based vaccine (ChAdOx1 and Ad5-NiVG), virus-like particles, and a recombinant rabies virus-based vaccine [94,95,96]. Vaccines that have been investigated for animals include a subunit vaccine for bats, pigs, and horses, and a canarypox virus-based vaccine (ALVAC) for use in swine [94,95]. A subunit vaccine for HeV based on the soluble glycoprotein (HeV-sG) was developed and made available for horses in Australia in 2012, and has been seen to be effective at inducing neutralizing antibodies [97]. To the authors’ knowledge, this is the only commercially available vaccine at this time. This success has led to a human vaccine candidate based upon the same principle (HeV-sG), which has demonstrated good results in animal trials (including non-human primates) and is entering human trials [98].

As has been discussed, initial NiV infections primarily occur as a result of spillover events from bats to animals (livestock such as pigs), which may then pass to humans or from bats directly to people (contaminated fruit and date palm sap) [1,37,91]. Thus, effective control measures should consider this transmission pathway, the ecology of bats as reservoirs, and the cultural and agricultural practices of people. As an example of this, it has been demonstrated that the use of nets over the trees helped to reduce the consumption of the date palm by bats, which prevented further contamination. Additional potential control measures could involve preventing the consumption of raw date palm sap where there is any potential risk of exposure to bats, by encouraging sterilization procedures, and banning the sale of raw products. Naturally, the success of these measures would be determined by the acceptance of local communities; thus, they should be accompanied by the extensive education and involvement of these communities.

Deforestation of natural habitats of bats is recognized as one of the drivers of increased interaction between bat populations and human civilizations.

Passive surveillance for Henipavirus antigen from wild bat populations and the guano from known nesting sites may be valuable to identify levels of virus circulating in the wildlife populations. Regions with active deforestation activities could be good surveillance sites, using an assay such as the SPB-ELISA, as mentioned in the text, with reagents that are not species-specific and thus better for broad surveillance [91].
